# Advancing inclusive science and systemic change: the convergence of national aims and institutional goals in implementing and assessing biomedical science training

**DOI:** 10.1186/s12919-017-0086-5

**Published:** 2017-12-04

**Authors:** Sylvia Hurtado, Damani White-Lewis, Keith Norris

**Affiliations:** 1 Higher Education and Organizational Change, Graduate School of Education and Information Studies, University of California, Los Angeles, CA USA; 2 General Internal Medicine-Health Services Research, David Geffen School of Medicine, University of California, Los Angeles, CA USA

## Abstract

**Background and purpose:**

National reports call for improving America’s leadership in scientific research, accelerating degree attainments, and diversifying the scientific workforce to foster innovation. However, slow progress and persistent disparities across growing U.S. populations are evident on key science workforce indicators, from degree attainment to career achievements. The purpose of this article is to provide a conceptual basis and overview of a national effort funded by the National Institutes of Health (NIH) that advances inclusive science practice and systemic change. We introduce the context, features, and rationale that drive practice and evaluation in the Diversity Program Consortium (DPC) approach, which is an experimental program to implement and evaluate evidence-based and novel practices to expand and diversify the biomedical workforce.

**Key highlights:**

Despite decades of federal investment for biomedical research training, researchers identified disparate adjusted rates of R01 grant awards by scientists’ race/ethnicity. This motivated NIH to fund the DPC approach as a set of highly integrated initiatives that empower institutional change agents to create scalable, evidenced-based strategies to enhance diversity in biomedical research and health science training. Key DPC elements include: 1) A systemic approach to enhance science preparedness involving students, faculty, and institutional-capacity development; 2) Collaboration, partnerships and networks across individuals and organizations, and especially between NIH, 10 undergraduate Building Infrastructure Leading to Diversity (BUILD) sites, the National Research Mentoring Network (NRMN), and the Coordination and Evaluation Center (CEC); and 3) Increased focus within and across key career stages for expanding training and ultimately diversifying the scientific workforce. A new framework for inclusive science practices and discussion of systemic change challenges provide insights into DPC processes and activities.

**Implications:**

Collectively, the DPC establishes a national learning collaborative to implement and evaluate multidimensional components of training and program interventions, accelerate the adoption of promising or effective practices, and disseminate lessons to the broader extramural scientific community. Linking practice with evaluation research will identify exemplars that others may adopt to advance the goals of inclusive science in promoting and sustaining innovation, accelerating equity in science careers and, ultimately, address challenging health problems in an increasingly diverse nation.

## Introduction

At the close of the World War II, a report to the President of the United States urged the federal government to “accept new responsibilities for promoting the flow of new scientific knowledge and the development of scientific talent in our youth…for they vitally affect our health, our jobs, and our national security” [1]. The Research Grants Office was created at the National Institutes of Health (NIH), shortly thereafter, to facilitate federal investment in a program of extramural research grants and fellowship awards [2]. National aims for advancing scientific research and training accelerated after the launching of Sputnik by Soviet scientists in 1957, an event widely recognized as the catalyst for expansion of the U.S. higher education system, federal investment in scientific research, and financial aid to college students to increase access to postsecondary institutions [3]. Today, the key areas of health, jobs, and national security remain critical foci that require investment, innovation, and the development of scientific talent at all levels of training. Because the majority of scientific research training occurs in postsecondary institutions that operate rather autonomously from the U.S. government, aligning national aims and institutional goals becomes essential. This is often accomplished through competitive funding for research and postsecondary training grants that reflect national priorities.

However, significant disparities across diverse populations from undergraduate education through early career stages called for a bold new approach to training and sustaining science productivity [4]. Persistent disparities across growing U.S. populations are evident on key science workforce indicators, from degree attainment to career achievements. For example, the National Science Foundation (NSF) reported that among doctoral degrees awarded in science, mathematics and statistics, engineering, psychology, and social sciences in 2012, women earned only 40%, blacks/African Americans 6.3%, and Hispanic/Latinos 6.5% [5]. Despite the existence of interventions, progress towards achieving a more diverse scientific workforce has been slow relative to the rapid increases in a demographically diverse U.S. population. The U.S. is projected to become a majority minority nation, with only 44% non-Hispanic whites by 2044 [6]. The purpose of this article is to provide an overview of the NIH-funded Diversity Program Consortium (DPC), which is a national effort to develop innovative models and institution-wide reform for biomedical training. We introduce the background, structures, key features and rationale that drive implementation and evaluation efforts to diversify U.S. biomedical science research training, as well as begin to define inclusive science and systemic change as it is reflected in practice.

## National Trends and background

Several key trends in the last decade set the stage for a national impetus for change and the development of the NIH-sponsored Diversity Program Consortium (DPC) as a renewed effort to address expansion and diversification of the biomedical research workforce. First, a succession of national reports documented the state of scientific training and research with calls to action. A widely-publicized 2007 report emphasized fading U.S. leadership in research and the production of STEM degrees, recommending increases in the percentage of all 24-year olds with first degrees in science to at least match the levels of other countries [7]. This resulted in legislation called the America COMPETES Act that included additional funding to support all scientific research and training programs. A subsequent Congressionally-mandated report in 2011 focused on reducing the severe disparities for underrepresented groups (URGs) that compose a growing share of the American population [8]. It recommended a comprehensive and coordinated approach, as well as financial assistance, to triple the number of URG graduates earning a first degree in natural sciences or engineering. Science advisors to the President also released a 2012 report that set new targets for a million more STEM graduates over the next decade [9], with a focus on aligning institutional practices that engage students to meet the national goal. Major associations also responded with their own calls for change to improve science teaching and faculty engagement with undergraduates [10, 11]. More recently, a 2016 National Academy of Sciences, Engineering and Medicine (NASEM) report documented the varied postsecondary educational pathways for diverse students and recommended “a series of interconnected evidence-based approaches to create systemic organizational change for student success” in science training [12]. These reports recommend government and foundation investment, and more importantly, coordinated institutional action to improve practices that address instruction, research training, and career mentoring to advance excellence and equity in the scientific workforce.

A related trend is that social science research has informed many of these national reports in terms of evidence-based practice, and strengthened the rationale for the value of diversity in science training and the biomedical workforce. Research on the benefits of diversity in the last 15 years identified the cognitive and democratic benefits of education with diverse peers for college graduates [13–15]. Benefits of diverse work environments have been documented in terms of innovation, creativity, and improved performance outcomes [16–19]. Collaboration on diverse work teams also results in higher impact publications, presumably due to broader perspectives that improve research quality and diverse networks that increase citations [20]. Social science research has also begun to unravel the accumulative processes that explain disparities perpetuated through hostile climates, implicit bias, and stereotypes, which affect decision-making and undermine talent development [21, 22]. Moreover, studies show that career advancement can be particularly stagnant for URGs without a strong network of mentors [23]. Mentoring plays a key role in the successful academic socialization of new faculty [23]. Social science research provides the evidence-based rationale for adopting more inclusive practices in science, increases awareness about the lack of progress, and ignites institutional reform.

## Origins of the diversity program consortium (DPC)

A prime example of how social science research spurs reform is the result of an NIH-funded study that served as a catalyst for introspection at NIH, plans for the DPC, and continuing discussion on diversity and disparities in scientific circles [24–26]. Ginther and colleagues focused on examining whether scientists of varying races and ethnicities, with comparable research records, have similar likelihoods of having awarded NIH R01 grant applications awarded [27]. The R01 mechanism is the oldest and primary way that NIH makes investigator-initiated grants, accounting for over half of all external research grant funding [27, 28]. Results indicate that applicants with good scores on their peer-reviewed proposals were more likely to be funded, but there were significant differences in award probability by race and ethnicity. After controlling for applicant’s educational background, country of origin, training, previous research awards, publication record, and employer characteristics, black applicants were 10.4 percentage points less likely to be awarded R01 grants compared to white applicants. Black and Hispanic investigators were also less likely to resubmit an unfunded application. The authors suggest that small differences in access to research resources and mentoring may result in a cumulative advantage for white investigators that creates large between-group differences, disadvantaging underrepresented investigators despite achievements that qualify them as principal investigators for a major research grant. This widely-publicized research served as an impetus for the formation of a working group of the Advisory Committee to the Director (ACD) at NIH, which reviewed the study and engaged in its own analysis to come up with recommendations to not only examine review processes but also level the playing field and address disparities across career stages [29]. Changes in URG disparities in award rates would require a more systemic approach to opportunity and talent development.

In June 2012, the Working Group on Diversity in the Biomedical Research Workforce (WGDBR), through the NIH ACD, provided concrete recommendations to improve the recruitment and retention of talented individuals underrepresented in biomedical research and prepare them for successful biomedical research careers [4]. The focus is on persons from underrepresented racial and ethnic groups, individuals with disabilities, or from disadvantaged backgrounds as identified in government statistics [30], and groups in the latest NSF report on *Women, Minorities, and Persons with Disabilities in Science and Engineering* [5]. The Working Group provided recommendations designed to evaluate issues related to a disparity in success rates for research grant (R01) applications between White and black applicants in review processes; and ultimately broaden participation of diverse individuals in biomedical fields using more systemic strategies that are transformative and sustainable. The recommendations set an NIH-wide priority to address new comprehensive strategies that include training, mentoring, and research engagement/support for students and faculty as well as resources for improving institutional capacity.

In one of many responses to WGDBR recommendations, the NIH launched a set of experimental programs in 2014 to implement novel and innovative initiatives to engage and sustain individuals underrepresented in biomedical research training and careers, while simultaneously building infrastructure and developing faculty that would support biomedical research activities [31, 32]. The multipronged approach was intended to sustain effective innovations introduced on and across campuses. Funded at $250 million for 5 years, this trans-NIH Common Fund [33] program established three highly-integrated initiatives: the Building Infrastructure Leading to Diversity (BUILD) initiative (RFA-RM-13-016) [31], the National Research Mentoring Network (NRMN) (RFA-RM-13-017) [34] and the Coordination and Evaluation Center (CEC) (RFA-RM-13-015) [35]. Collectively, the BUILD, NRMN, and CEC grantees work together with the NIH as the Diversity Program Consortium (DPC). Throughout the funding period, grantees implement local intervention strategies as well as a consortium-wide approach to assessing key Hallmarks of Success as outcomes for students, faculty and institutions. The NIH uses a cooperative agreement funding and management mechanism (U54 and UL1) that involves substantive scientific involvement of the grantee in collaboration with NIH. The DPC establishes a national learning collaborative to investigate the multidimensional components of training and program interventions, accelerate the adoption of promising or effective practices, and disseminate lessons NIH-wide and to the broader extramural community.

## Components of the diversity program consortium (DPC)

**Fig. 1:**
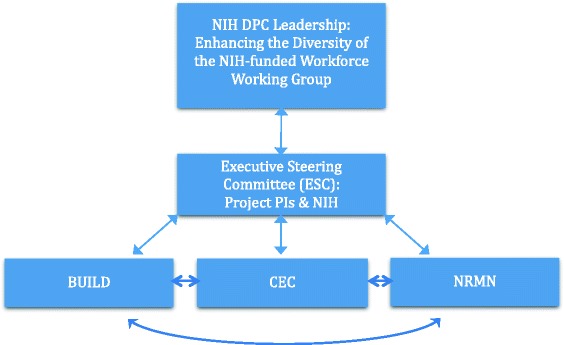
The Diversity Program Consortium (DPC)

Figure 1 shows the structure of the DPC in terms of leadership and the key components of the initiative. The NIH DPC leadership is comprised of the NIH Chief Officer for Scientific Workforce Diversity and several Institute Directors who provide executive oversight to the Enhancing the Diversity of the NIH-Funded Workforce Working Group that involves members who bring unique perspectives based on their respective NIH roles and responsibilities [36]. The vision of the NIH DPC leadership group is implemented through the National Institute of General Medical Sciences (NIGMS), various NIH project officers and scientists, and the leadership of the grantees representing BUILD, NRMN and CEC who together form the DPC Executive Steering Committee (ESC). The DPC ESC represents the consortium interests and works as the operating body for DPC policies and procedures. It is the formal conduit for each grantee to other grantees and the NIH for consortium related needs and concerns.

In addition to the BUILD, NRMN, and CEC programs, the Advisory Committee to the Director (ACD) Working Group on Diversity (WGD) was formed in response to the WGDBRW recommendations [4] (not shown in Fig. 1). As a permanent working group, the ACD WGD provides advice to the ACD and, in turn, the NIH Director about programs such as the DPC and “effective strategies to increase the representation of diverse individuals underrepresented nationally in biomedical research and to reduce disparities in research awards from diverse applicants underrepresented nationally in biomedical research” [37].

### BUILD

To support the design and implementation of innovative training programs and strategies to promote scientific workforce diversity, the NIH established the comprehensive BUILD initiative to transform three synergistic areas that influence student achievement: student development, faculty development, and institutional capacity-building. Ten undergraduate institutions received BUILD grants that are funded as three linked awards associated with the BUILD Cores: a UL1 Linked Specialized Center Cooperative Agreement award to support Administrative and Institutional Development Cores; an RL5 Linked Education Project award to support the Research Enrichment Core; and a TL4 Linked Training Award to support the Student Training Core (see description of components [31]). BUILD is designed to implement and assess novel projects to improve undergraduate recruitment and retention of student talent in biomedical fields. BUILD also provides opportunities for alterations and renovations of laboratory and training facilities, curriculum development, pilot project research funding, mentor-mentee activities, and early student engagement in hands-on-research. BUILD grantees may partner with pipeline, research-intensive, and graduate/medical academic institutions, as well as industry to strengthen research training, promote faculty collaborations, and expand the pool of students engaged in BUILD activities (see participating institutions link [38]). Leadership structures of the programs are variable, consisting of single and multiple principal investigators (PIs), often including a provost or administrative official as PI or multiple PIs from both the primary grantee and major partner institutions. Successful BUILD programs are expected to yield tangible advances in student, faculty, and institutional development; and through its multifaceted funding model, the program is also expected to transform and institutionalize biomedical training environments for currently enrolled students and those following them for years to come.

### NRMN

Recognizing that the lack of mentoring was a persistent problem at all careers stages, from undergraduate education to entry into the professions to senior leadership roles, NIH funded the development of a national network of highly motivated and skilled mentors from various disciplines to be linked with mentees across the country. NRMN is charged with developing best practices for mentoring, training opportunities, and professional development for mentees and mentors. One award went to a group of five academic institutions that coordinate activities in regional hubs across the country, collaborate to provide services, coach faculty in grantwriting and to take on effective mentoring work, introduce novel approaches (e.g. culturally aware mentorship, virtual-guided mentorship), and produce scholarship on mentoring that can be widely disseminated [39, 40]. According to the funding announcement (RFA), expectations are for NRMN to help establish Hallmarks of Success [41], develop standards and metrics for effective face-to-face and online mentoring, connect individuals with experienced mentors in face-to-face and through online networks, develop and test innovative mentoring strategies, engage in active outreach to those with limited access to research mentors, teach effective mentoring skills, provide professional development activities, enhance access for mentees of diverse backgrounds to learn about biomedical careers and funding opportunities, and ultimately enhance their ability to attain NIH funding (see RFA-RM-13-017) [34]. NRMN grantees are expected to develop and implement sustainable practices that can go beyond the life of the grant. The leadership structure for NRMN works though an Administrative Core that manages and coordinates with those leading several task-specific Cores on Mentorship and Networking, Mentor Training, and Professional Development. Specifics regarding NRMN developments, programs, and events can be found at the DPC websites of NIH and NRMN.

### CEC

The NIH funded a Coordination and Evaluation Center (CEC) to coordinate Consortium-wide operations and facilitate the development of consortium-wide goals, create design instruments, conduct robust evaluation of measures of success toward the individual and consortium-wide goals, and serve as a focal point for consortium communication and dissemination [35]. The NIH recognized that, in contrast to traditional monitoring of trainee success, a more extensive evaluation would be needed to better understand the impact of the Consortium activities. Although there are other NIH-sponsored national longitudinal studies of URG science training [42, 43], on behalf of the DPC, the CEC leads a unique hypothesis driven, large-scale systemic national longitudinal evaluation of research training programs. Using both established and newly developed common measures across sites, and mixed quantitative (e.g. rigorous quasi-experimental designs) and qualitative methods, the CEC focuses on long-term outcome assessment and identifying scalable transformative approaches that have potential for a broad and sustained impact on the diversity of the biomedical research workforce. Key to the comprehensive evaluation of the impact of the DPC was the creation of agreed upon evidenced based and novel consortium-wide Hallmarks of Success, which are embedded in BUILD and NRMN logic models that align expected outcomes with practices focused on critical training and career transition points. The CEC leads the development of high-impact dissemination strategies to inform peer institutions, NIH, and other key national and international stakeholders about results from the implementation of practices at the student, faculty and institutional levels. In summary, the CEC provides coordination and technical support for Consortium-wide activities including the processes for designing and managing data collection, conducts a rigorous longitudinal evaluation across programs, and provides a framework for the assessment of factors common to interventions that lead to the success of well-trained individuals from diverse backgrounds in biomedical research, with national implications for scalability to other institutions and research training programs.

## Key features of the DPC approach

It is important to note that the NIH has long recognized an urgent and compelling need to address diversity in the biomedical research workforce. Programs under the NIH-NIGMS Division of Training, Workforce Development, and Diversity (TWD) have focused on diversity in undergraduate research training as early as 1972 [44]. The DPC is not intended to replicate existing programs but implement new ones in the context of an array of programs that are significant investments to engage scientists using training and mentoring approaches for undergraduates, postdoctoral scholars, and faculty. These previously existing NIGMS training programs have resulted in positive outcomes for trainees and participants [45, 46]. However, a new approach to implementation and evaluation was needed because reports focused on outcomes have not identified socio-psychological, practice, or institutional factors in success that can be institutionalized and scaled for widespread dissemination. The DPC and the key features of this historic collaboration for advancing diversity in biomedical science training are described in the sections that follow.

### Collaboration as the mode of operation

Various forms and levels of collaboration are essential aspects of a working consortium. The first level of collaboration within the DPC is intra-institutional; for example, within BUILD campuses institution-wide support is reflected in the involvement of multiple campus units (i.e. academic departments and varying levels of administrative authority). Most DPC grantees also have interdisciplinary initiatives involving faculty collaborators and trainees in the biosciences, engineering, public health, and the social and behavioral sciences that address research on biomedical topics, health science and disparities, and/or expertise in science education. Inter-institutional collaborations among DPC grantees involve partner institutions in the region, as well as with industry or other NIH grantee institutions, to increase research opportunities for students and faculty. While BUILD sites may be regional hubs of collaborative activity, the growing network that is NRMN is built upon multiple institutions and national involvement of scholars, coaches, mentors, and mentees. Collaboration is also inter-organizational, primarily between NIH and DPC grantees, as funding is dependent on cooperative agreements that are managed in close consultation between organizations. Collaboration between Consortium members requires regular communication between groups including monthly meetings and webinars, monthly or bi-monthly working groups that discuss evaluation, program recruitment, and communications and an annual meeting of grantee teams. Common outcomes are articulated in a consortium-wide adopted document reflecting Hallmarks of Success [41], data sharing agreements, and planning documents. This is in contrast to other training grants, for example, where PIs implement programs according to general RFA guidelines and reporting requirements, often working independently within an institution or with limited collaboration outside of the grantee institution. The primary exception is if the training grant calls specifically for collaboration between institution types, in which case, there can be successful outcomes across shared resources [44]. The DPC depends on multiple types of collaboration for success and has a shared purpose, works on problems that arise and then reaches consensus to forge directions.

### Evaluation is central

The DPC initiative incorporates a rigorous evaluation from planning through implementation to assess the efficacy of its programs, which addresses challenges and concerns cited about previous NIH training programs that lacked clear, measurable training goals or a rigorous evaluation [47]. The DPC stimulates new creative approaches for training and mentoring interventions for individuals with a broad range of biomedical and behavioral research interests, and is providing data to “help shape and revise current diversity training programs and inform new ones” [32]. That is, evaluation was made central to the DPC initiative, implemented on both a national scale coordinated by the CEC for determining what works in terms of best practices and sustainability as well as local evaluations for refining BUILD and NRMN program activities (see each article in this supplement volume for evaluation methods and innovations).

### Evidence-based practice and novel approaches are implemented

National reports urging improvement in science training relied on advances in federal data collection to monitor statistics and disparities across groups [5, 48], but also depended on a growing body of social science research on science education [49, 50], and evidence-based practice in teaching [51]. From 2004 to 2013, NIH invested in a specific R01 program to advance an understanding of the science behind training interventions that now inform many of the initiatives embedded in DPC activities (e.g. see for example Research on Interventions projects on mentoring, undergraduate research, minimizing bias and stereotype threat in STEM) [52]. Thus, practices in science training have a more substantial research base, and DPC programs are expected to implement evidenced-based approaches as well as novel approaches that can extend the evidentiary basis for practice. All aspects of this grand experiment may serve as an impetus for reform within institutions and implementation strategies across institutions to enhance diversity in the biomedical workforce.

### A systemic approach involves multiple career stages, comprehensive activities, and shared resources

Across the DPC, activities span multiple career stages. For example, NRMN facilitates access to mentoring relationships and provides coaching activities for participants across multiple career stages and disciplines [39, 40]. Instead of focusing on a single PI’s program, BUILD funding goes to specific institutions to foster institutional capacity and accelerate degree productivity among URG students. Expectations are that grantees develop comprehensive initiatives that address and assess both academic and psychosocial factors that are well-known to influence why individuals persist in science [53–56]. BUILD activities are required to develop students’ skills and learning dispositions and ultimately science identities, faculty skills in research and training, and institutional capacity for addressing biomedical research and training. The latter involves, for example, acquisition of advanced technical instruments, new positions to facilitate research training, and classroom renovations for active learning. With substantial institutional investment, senior administrative leaders are expected to support BUILD initiatives and encourage institution-wide buy-in to sustain practices. BUILD also partners with high schools or community colleges (or pipeline partners) to identify and recruit early talent that matriculates into their four-year institutions. They also link with research-intensive partners to increase research opportunities, train participants using state-of-the-art equipment, place students in graduate school, and encourage faculty research collaborations. A systemic approach is intended to recognize and strengthen diverse student pathways in biomedical fields, encourage shared resources, and increase research opportunities for talent development at all levels of biomedical training.

## Diversity, equity, and the concept of inclusive science

Before describing how diversity and equity is embedded in practice across the DPC, it is important to note that rather than fund institutions that already have significant extramural resources, the NIH targeted BUILD funding to undergraduate institutions that serve highly diverse student populations and have the capacity to accelerate the production of biomedical researchers. Specifically, institutions eligible to apply were those that received less than $7.5 million (total costs) of NIH research project grant funding annually and served an undergraduate student body where at least 25% are supported by federal grants for low-income students (i.e. Pell grants). “These requirements are based on the recognition that (1) many students from low-income backgrounds are also nationally underrepresented in biomedical research, and (2) institutional commitment to these students often comes at the expense of investments in research infrastructure” [31]. The BUILD institutional grant encourages activities that both reinforce their commitment to student success and increases institutional capacity for research in ways that will expand opportunity for diverse faculty and students. Many of the primarily undergraduate teaching institutions benefit from improving their own research capacities, as well as partnering with research-intensive institutions, to leverage resources and opportunities for their faculty and students. A unique aspect of many BUILD institutions is the development of partnerships with many of their key pipeline institutions (e.g. community colleges, high schools) to help enhance their STEM curricula and research opportunities that will better prepare and position aspiring students for successful biomedical research careers.

This targeted institutional approach raises an important question, how does one think about diversity when a campus already has a diverse student population? Minority serving institutions (historically black institutions, Hispanic serving institutions, and tribal colleges) are often cited as the source of a large proportion of URG graduates who pursue science or advanced degrees [57, 58]. Those with a historic focus on underserved populations have an institutional ethos on the advancement of these communities. This is not the case, however, with many predominantly white institutions that have recently increased demographic diversity and must rely on interventions to help URG students navigate courses and research hurdles [59]. Campus-based research shows disparities when educational outcomes are disaggregated by race, ethnicity, gender, low-income, or first generation status within diverse as well as in predominantly white institutions [54, 60, 61]. One can look at such numbers from a deficit perspective or begin to use an equity-mindset [60], focusing on equity in outcomes as a relevant accountability metric to monitor progress on demographically diverse campuses to determine if gender or racial groups, for example, have an equal probability of success in biomedical majors. Low diversity in the faculty and few women in advanced faculty ranks also remains an equity issue that affects all campuses [62], and much underrepresentation is a result of unequal opportunity at earlier career stages. In addition to using an equity perspective, a second way to think about diversity is to consider how training activities translate into inclusive practices that address culturally diverse and underrepresented populations.

“Diversity means an inclusive approach, both to the science itself and the make-up of the groups of people who carry out the research” [24]. Research indicates, however, that improving compositional diversity is a necessary but not sufficient condition to achieve the benefits of diversity; and while one can have a demographically diverse science classroom, for example, it can still lack the pedagogy that engages students in ways that takes into account their social identity and lived experiences, or creates interactions among peers that result in educational benefits [15]. The concept of inclusive science has been previously introduced in reference to classroom-level innovations, yet is specifically limited to those that are “designed to promote the inclusion of all students in science [that] range from those identified specifically as female-friendly or culturally inclusive, to those that encompass both gender and culture” [63]. An inclusive approach to science involves a renewed appreciation for all forms of diversity, calls for understanding and respect for the contributions of people from different backgrounds, and a reconsideration of assumptions about URGs that are often the target of bias and stereotype in activities that occur both inside and outside of the classroom, as well as in professional contexts. While inclusive science is, in part, a culturally responsive approach we extend the context and dimensions of inclusive science practice using emergent practices within the DPC.

**Fig. 2:**
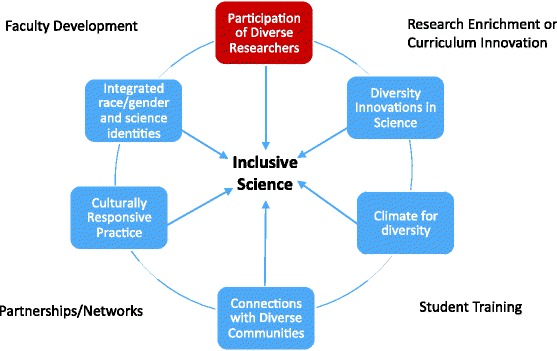
Dimensions of Inclusive Science Embedded in Functional Areas of Practice. Note: Functional areas are major components of grantee activity and practice implementation that coincide with funded Core or areas stipulated in grant proposals. BUILD and NRMN differ in foci, and the general model of practice does not reflect infrastructure or Institutional or Administrative Core funded activities

Change agents across the DPC are actively redefining the concept of inclusive science, addressing both diversity and equity in biomedical training practices to advance scientific research. We introduce a model of conceptual areas (in Fig. 2) that further define an inclusive approach to science. Using examples across the DPC, we illustrate approaches to inclusive science embedded in many practices that can be implemented at other campuses and in biomedical science training activity. The framework involves not only compositional diversity of researchers, but also diversity innovations in science, climate campus improvement, diverse community partnerships, revision of practices to become more culturally responsive, and exposure to role models that integrate science identity and URG’s social identities. The interrelated dimensions of the framework may be refined over time as successful practices are identified and new ways of thinking and practice emerge. (For more context specific information of each example in this section, see the corresponding article on site-specific practices in this volume).

### Participation of diverse researchers

A central goal of an inclusive science approach is to develop a cadre of skilled researchers and reduce disparities in representation across fields of study and career stages. A primary goal of the DPC is to develop researchers at all career stages, ranging from college entry to early career attainments, to eventually become part of the NIH-funded workforce. This is accomplished by providing opportunities to individuals who may not typically have access to research mentors or comprehensive support for success in biomedical science fields. For example, the University of Maryland, Baltimore County (UMBC) has a history of a highly successful program for high achieving URGs in the Meyerhoff Scholars program [64]. Their BUILD initiative is focused on increasing the number of trained biomedical graduates through reform of curricula, expansion of training opportunities, and the inclusion of more students who may not apply or qualify for existing scholarship programs at admission but could benefit from talent development support in college. Xavier University of Louisiana has a history of advancing African American graduates into graduate and professional schools, with a number of successful extramurally supported programs to advance students in biomedical fields. Their BUILD initiative is focused on coordinating all programs that result in a developmental sequence of research experiences throughout the college years that includes intensive, tailored career and academic advising. A post-baccalaureate opportunity is also offered for students who missed research opportunities during their undergraduate years and/or lack research experience and preparation needed for graduate school. Both campuses, and other BUILD sites, have adopted a talent development approach and increase opportunities in biomedical fields to increase the number of graduates. At later career stages, NRMN is expanding the network of mentors and mentees engaged in biomedical research, and grantwriting coaches help to address underrepresentation in NIH-funded extramural research.

### Diversity innovations in science

One way to increase the number of diverse researchers is to address topics and issues in science that involve discovery and advancing knowledge about demographically diverse communities. An inclusive science approach reflected as diversity innovations in science may involve novel curricula, new research questions that address health issues that impact specific demographic groups, new research methodologies for marginalized population research, or new knowledge resulting from interdisciplinary collaborations. For example, California State University (CSU), Long Beach promotes early and well-trained researchers to approach major health challenges in a multi-disciplinary way and incorporates this into the curriculum and research communities. University of Alaska, Fairbanks (UAF) has adopted a One Health initiative that involves a focus on the important links between the health of humans, animals, and the environment in a series of courses that also involves faculty from multiple disciplines. This thematic and problem-based approach to novel curricula is particularly relevant to rural communities, which are primarily indigenous, and can also lead to further innovations in science. These approaches not only involve a culturally responsive approach (described in a subsequent section) but also open the possibility for new knowledge that result from authentic research on relevant topics (i.e. as a result of studies using new methods and multiple disciplines to address the health of underrepresented communities). Building on URG students’ prosocial values in career goals, individuals may be attracted to and retained in science if they can understand alignment with their own values or relevance to their communities while engaged in discovery of new knowledge [54, 65, 66].

### Climate for diversity

Advancing an inclusive approach to science involves improving the climate in classrooms, labs, and interactions on campus. For example, perceptions of a hostile and competitive climate are associated with lower sense of belonging and adjustment to college among aspiring biomedical scientists [67]. The climate also affects students’ sense of belonging in STEM that, in turn, is associated with higher satisfaction and better performance [68]. A number of efforts seek to improve the climate for diversity by educating faculty about marginalization and microaggressions, which are “brief, everyday exchanges that send denigrating messages to people of color because they belong to a racial minority group” or other non-dominant group [69]. For example, CSU Northridge BUILD conducts faculty development using Critical Race Theory to help faculty understand racism and to recognize and diminish microaggressions in daily interactions on campus [70]. The campus focuses on providing a counterspace for a community of equity-conscious faculty and student researchers to address challenging health disparity issues. CSU San Francisco and campus partners are committed to creating intellectually safe and affirming environments to reduce the consequences of stereotype threat (i.e., a phenomena where persons fear conforming to stereotypes about their social group, such as low expectations of academic success for women and minorities), which can affect student performance and retention [53, 71]. NRMN improves the climate in laboratories and professional environments by training faculty to adopt more effective mentoring practices, and empower mentees in various contexts.

### Partnerships with diverse communities

Inclusive science may also involve direct contact with diverse communities that have been previously invisible in data or programs, or marginalized due to location and lack of opportunity. For example, Portland State University (PSU) BUILD partnerships involve students and institutions located throughout Oregon, the Pacific Rim, and Pacific Northwest, which include indigenous and underserved communities that are often isolated and have few research training opportunities. Similarly, the University of Alaska Fairbanks (UAF) is building partnerships with campuses, students, and communities in remote areas of Alaska that still rely on natural resources and subsistence economies integrated with cultural traditions. ReBUILD Detroit is composed of an integrated, cross-institutional teaching and learning community for undergraduates and faculty among higher education institutions in an urban setting moving toward economic recovery. Community partnerships on local issues and problems become the focus of classroom and research activity to improve the health of students’ home communities. Other BUILD campuses engage communities by involving educators, parents, and local community organizations to identify and support talented and aspiring biomedical scientists.

### Culturally responsive practice

DPC practices are helping to define culturally responsive practices in science contexts. Culturally responsive practice is most often referred to when discussing pedagogy or “teaching practices that embrace the whole student in the learning process” and provides “insight into how college educators can create classrooms in which diversity is valued” [72]. It is important to note that BUILD campuses demonstrate an awareness of their students’ concerns and needs as low-income, first-generation, or URGs in science. For example, University of Texas-El Paso is largely an open admissions institution located near the Mexico border. This BUILD institution has adopted an asset-based approach to science training [73], helping students to recognize their strengths and preparing them for engagement in authentic research and presentation opportunities as early as the first year in college. CSU Long Beach encourages faculty and staff to recognize their students’ resilience and cultural strengths, and emphasizes a growth mindset. Culturally responsive practice can also be reflected in mentoring contexts. To address this NRMN has created a module on culturally aware mentoring to encourage critical self-reflection and help mentors recognize their own and mentee’s cultural identity and worldviews as it may affect interactions in research training [39].

### Integrated science identities

A related element are programs that provide opportunities and role models that encourage the integration of participants’ identities as aspiring scientists and researchers with their social identities that constitute authentic selves. Research on women of color in STEM has led to the development of a science identity model (composed of competence, performance and recognition from significant others) that is shaped by race and gender [74]. DPC participants are engaged in evaluation regarding science identity and how it may vary across time or career stage in programs. For example, Morgan State University’s ASCEND program emphasizes student ownership of knowledge production, sense of belonging, and the development of students’ science identity within the context of a historically black university. Opportunities to integrate identities and interact with diverse researchers as role models can also be found in activities such as the meeting of the Society for the Advancement of Chicanos and Native Americans in Science (SACNAS), the Annual Biomedical Research Conference for Minority Students (ABRCMS) and local science professional clubs. These activities allow participants to demonstrate competence, practice performance, and obtain recognition from peers and faculty. The DPC has held program-wide meetings that coincide with the SACNAS annual meeting of students, postdoctoral scholars, and faculty to celebrate the scientific accomplishments of diverse researchers.

## Systemic change challenges and prospects for the future

Many of the practices in the Inclusive Science model cannot be enacted without an institutional opportunity to experiment, willingness to engage in systemic and institutional change, and broad institutional buy-in. Much will be learned about how to enact institutional change from DPC grantees, collaborating partners, and working groups. It has required energetic champions to move the vision of change forward on all sites, relationship-building, and some amount of risk-taking to attempt a new approach because it involves a critique of how typical ways of teaching, mentoring, and research may no longer be suitable for the twenty-first century needs [75]. Changing the norms of an institution can be hard work if one is confronted with daily forms of resistance from other faculty, specific departments, or institutional leaders who lack awareness or may passively oppose inclusive or evidence-based practice. Each change agent involved in DPC activities has had to develop allies, advocate for change, recruit support from colleagues, and strategize to make initiatives successful. They developed innovations alongside traditional teaching and training practices, navigated institutional policies, and have recruited individuals that are critical to the success of initiatives. Extramural support has helped facilitate and increase attention to change efforts but, without a growing number of participants willing to contribute on each site, the investment does not guarantee buy-in from all segments of campus or partner institutions needed for sustainability.

Moving from a focus on a single intervention biomedical training approach that served small numbers of scholars to a whole trainee, whole institution approach has been a significant scale-up challenge locally and nationally in the DPC. Many of the BUILD sites are committed to providing broad access and serving students with a variety of needs. It has been a challenge to simultaneously take on intensive student development interventions and new faculty development interventions, for example. Institutions have multiple aims and campus cultures (e.g. academic culture, business culture of the administration, student cultures) and methods to advance their teaching, research, and service missions. The realities of high teaching loads and raising research capacity at BUILD campuses has created new tensions that must be resolved and may begin to reshape institutional identity. Valuing skilled mentorship and training is integral to individual success and program outcomes, for example. These new duties that come about as a result of the DPC have had implications for faculty workload and rewards. Faculty and staff contributions to national goals to enhance the diversity of the biomedical workforce should be reported and valued as evidence of impact in tenure, promotion, and merit reviews.

Many DPC grantees have been used to working independently as PIs of research and training grants. While a few had established working collaborative relationships before the DPC, the multiple levels of collaboration described as a key feature of the Consortium has been time-consuming, involving multiple institutions, people, and organizations. In a way, it has worked according to the same principles as described in the literature regarding how diversity works [14, 17], requiring each party to step out of their comfort zones, learn from each other, and work in interdisciplinary groups and diverse teams. It has required mutual respect for expertise and willingness to hear each point of view to focus on improving an implementation strategy or evaluation procedure with quality results. In the end, an enormous amount of respect and appreciation has resulted from understanding the level of commitment and hard work that each party has devoted to the success of local initiatives and to the DPC as a whole. Old networks have been expanded and new networks have been established as a result of DPC collaborative activity.

Although DPC grantees have been engaged in inter-institutional partnerships, changes in terms of mentorship training and program philosophies or approaches are not always part of the repertoire of training strategies at many partner institutions, especially at research-intensive universities. Finding ways to diffuse novel approaches and philosophies to more scientists and institutions, especially those dependent on extramural NIH funding, will be critical to creating wide-spread and lasting progress in enhancing diversity in the biomedical workforce. While NRMN has been making headway at research-intensive institutions to improve mentorship, DPC trainees will likely enter graduate school and work in environments driven by old training models and philosophies. The extent to which DPC initiatives take hold in other institutions is still unknown, but the hope is that trainees emerging from the experience are likely to pay it forward in terms of leadership, innovations in science, and awareness— serving as the next generation champions of excellence in research and inclusive science. The nations’ health, jobs, and biomedical-related security priorities will depend on their success.
